# Fragility Fractures in End-Stage Chronic Kidney Disease (CKD) Population: Patient-Related and CKD-Related Factor Analysis—A Single-Center Experience

**DOI:** 10.3390/jcm13082430

**Published:** 2024-04-21

**Authors:** Domenico De Mauro, Gianmarco De Luca, Silvia Marino, Amarildo Smakaj, Giuseppe Rovere, Francesco Liuzza, Marcello Covino, Pierluigi Fulignati, Giuseppe Grandaliano, Omar El Ezzo

**Affiliations:** 1 Orthopedics and Traumatology Unit, Department of Ageing, Orthopedics and Rheumatological Sciences, Fondazione Policlinico Universitario A. Gemelli IRCCS, L.go A. Gemelli, 8, 00168 Rome, Italy; 2 Department of Orthopedics and Geriatric Sciences, Catholic University of the Sacred Heart, 00153 Rome, Italy; 3 Orthopedic Unit, Department of Public Health, “Federico II” University, 80138 Naples, Italy; 4 Nephrology Unit, Department of Medical and Surgical Sciences, Fondazione Policlinico Universitario A. Gemelli IRCCS, 00168 Rome, Italypierluigi.fulignati@policlinicogemelli.it (P.F.);; 5 Orthopedic and Traumatology Unit, “Tor Vergata” University, 00133 Rome, Italy; 6 Department of Emergency, Anesthesiological, and Resuscitation Sciences, Fondazione Policlinico Universitario A. Gemelli IRCCS, 00168 Rome, Italy

**Keywords:** fragility fractures, chronic kidney disease, dialysis, epidemiological study, mineral bone disease

## Abstract

**Background:** Chronic kidney disease (CKD) stands as a prevalent global health concern, and mineral and bone disease are among the most impactful consequences. A severe complication arising from mineral and bone disease is the occurrence of fragility fractures, which disproportionately affect individuals with CKD compared to the general population. The prevalence of these fractures impacts both survival rates and quality of life. The aims of this study are analyzing and identifying (i) patient-related risk factors and (ii) CKD-related risk factors to contribute to the development of preventive measures for fragility fractures for this population. **Methods:** A retrospective, single-center observational study was conducted, encompassing patient data from the years 2021 to 2023. Registry data were recorded, including patient-related and CKD-related data. Patients were interviewed about traumatological history, and their answers were recorded. Logistic regression analysis was employed to investigate the association between independent variables and dependent variables. **Results:** Eighty-four patients, with a mean age of 64.3 ± 15.2 years and a male percentage of 58.3%, were included in this study. Among them, 19.5% exhibited smoking habits. The mean Charlson Comorbidity Index was 3.06 ± 1.21. All patients were diagnosed with end-stage chronic kidney disease, with mean durations of 208 months from the diagnosis and 84.5 months from the beginning of dialysis. The logistic regression analysis, adjusted for age, sex, and CCI, revealed that smoking habits play a significant role as a risk factor for fragility fractures in lower limbs (*p:* 0.011 *). The incidence of fragility fractures increases directly proportionally to the time since diagnosis (*p*-value: 0.021 *) and the beginning of dialysis treatment (*p*-value: 0.001 *). **Conclusions:** Among patient-related factors, smoking habits seem to significantly affect lower-limb fracture rates (*p* < 0.05), whereas among CKD-related factors, time since CKD diagnosis and time since the beginning of dialysis treatment are directly related to higher risks of fragility fractures. No relevant correlations emerged in the studied treatments, except for a reduction in proximal femur fracture occurrence when patients underwent a combined treatment of a calcimimetic and a vitamin D analog.

## 1. Introduction

Chronic kidney disease (CKD) stands as a prevalent global health concern, marked by the gradual deterioration of renal function over time, giving rise to various complications [1,2]. Among these complications, mineral and bone disease emerge as some of the most impactful consequences [3]. This is a consequence of abnormalities in the vitamin D, parathormone (PTH), Fibroblast Growth Factor 23 (FGF23), and calcium and phosphorus metabolism [4].

The initial signs of mineral and bone disease become evident with changes in calcium and phosphorus levels, coupled with an elevation in PTH levels, typically occurring when the glomerular filtration rate (GFR) falls below 40 mL/min (G3 stage) [5]. However, certain alterations, such as increased FGF23 levels, vascular calcification, and disruptions in bone formation rates, may manifest even earlier in the progression of this disease [6].

One of the kidney functions is the conversion of 1-hydroxy-calcitriol into the active form 1-25-dihydroxy-calcitriol. Fibroblast Growth Factor 23, inhibiting the 1-alpha-hydroxylase enzyme [7], and the progressive loss of kidney function precipitate a lowering of calcitriol levels and an increase in the levels of phosphate, promoting, in turn, an increase in Fibroblast Growth Factor 23 levels [8]. All these factors contribute to the hypocalcemia triggering the positive feedback for the release of PTH, which has the function of reabsorbing the calcium ions from bones and promoting phosphate excretion, leading to mineral bone disease [9,10,11].

A severe complication arising from mineral and bone disease is the occurrence of fragility fractures, which disproportionately affect individuals with chronic kidney disease compared to the general population. A fragility fracture is defined as a fracture that occurs in the absence of a traumatic mechanism or is brought about with minimal force that typically would not result in a fracture, as per the latest guidelines from the World Health Organization (WHO) [12,13]. The prevalence of these fractures significantly impacts both survival rates and overall quality of life for CKD patients [14,15,16]. Beyond the health implications, the socio-economic burden is substantial, evident in elevated healthcare costs and adverse consequences for affected individuals. Thus, it is imperative for modern healthcare systems to prioritize the prevention of fragility fractures among CKD patients [17,18,19]. 

The treatment in this regard is based on the correction of phosphate levels via dietary corrections or the use of phosphate binders, the correction of vitamin D values using vitamin D analogs, and the correction of PTH levels; in the last case, Calcitriol is usually used as the first-line treatment, but it could be ineffective if the PTH levels are very high, so KDIGO recommends combination with a calcimimetic (cinacalcet or etelcalcetide) [20]. 

Aims of the study are: (i) analyzing and identifying patients-related risk factors in determining fragility fractures in end-stage CKD patients, and (ii) analyzing and identifying CKD-related risk factors, such as disease history and progression and current treatment, to contribute to preventive measures for fragility fractures in this population.

## 2. Materials and Methods

A retrospective, single-center observational study was conducted, incorporating patient data from the years 2021 to 2023. This study adhered to the principles outlined in the Declaration of Helsinki. The Local Ethics Committees reviewed the study protocol and determined that no ethical approval was required, given the purely retrospective and observational nature of the study design.

### 2.1. Demographic Data and Inclusion/Exclusion Criteria

Through systematic database research conducted on the institutional registry, patient data were meticulously collected and recorded to satisfy the study’s objectives. This included collecting a comprehensive set of personal information such as age, gender, clinical and surgical history, diagnosis details related to chronic kidney disease (CKD), blood analysis results, pharmacological treatment, and the details of nephrological follow-ups. Data regarding the initiation of dialytic treatment and time since CKD diagnosis were also collected. The inclusion criteria comprised individuals with (i) a diagnosis of end-stage CKD necessitating dialytic treatment, (ii) age > 18 years, (iii) having suffered fractures characterized as fragility fractures as per the latest definition in the literature, and (iv) the ability to independently respond to the questionnaires administered. Conversely, exclusion criteria encompassed cases with (i) incomplete data, (ii) a history of tumors, (iii) fractures after subjection to high-energy trauma, and (iv) vertebral fractures.

### 2.2. Collected Data and Questionnaire Administration

Each patient who had been undergoing dialysis at our institution’s dialytic center participated in answering a questionnaire administered by doctors from the nephrology and orthopedics departments, who were also responsible for constructing the questionnaire. The questionnaire was administered prior to the dialytic procedure, and patients were asked about their history of previous fractures, fractures occurring since they developed end-stage CKD, and, in more detail, the number of fractures involving the upper and lower limbs, pelvis, and spine. The questionnaire further inquired about the specific segments affected, ranging from the proximal to distal regions.

Each response was carefully collected and entered into a pre-existing database, which incorporated data from the institutional registry. In addition to the previously mentioned information, personal details such as smoking habits and alcohol consumption were included. The Charlson Comorbidity Index (CCI) was also recorded for each patient, providing a comprehensive dataset for our analysis.

### 2.3. Statistical Analysis

The data are expressed as means and standard deviations for continuous variables, while frequency distribution (%) is employed for categorical variables. In the statistical analysis, we utilized the Student’s *t*-test for continuous variables and Chi-squared and Fischer’s exact tests for dichotomous variables. Logistic regression analysis was employed to investigate the association between independent variables and dependent variables. Potential confounding variables, identified through a literature review and theoretical considerations, were included in the models to control for their effects. The assumptions of linearity, independence, homoscedasticity, and normality were assessed for each regression model. Significance was set at *p* < 0.05. The statistical analysis was conducted using the SPSS software program 29.0 version (SPSS, Inc., Chicago, IL, USA).

## 3. Results

Eighty-four patients, with a mean age of 64.3 ± 15.2 years and a male percentage of 58.3%, were included in this study. Among them, 19.5% exhibited smoking habits, and 28% of the population had a concurrent diagnosis of diabetes. The mean Charlson Comorbidity Index value was 3.06 ± 1.21. All patients were diagnosed with end-stage chronic kidney disease, with a mean duration of 208 months since the diagnosis and 84.5 months since the beginning of dialysis. Regarding the current treatment for mineral bone disease, 60.7% of the population received a vitamin D analog. Within this group, 38.1% took calcitriol (1,25-dihydroxycholecalciferol, the active form of vitamin D_3_), while 23.8% used paricalcitol (an analog of 1,25-dihydroxyergocalciferol, the active form of vitamin D_2_). Calcimimetics, on the other hand, were employed by 35.7% of the patients, with the specific choices being 1.2% Cinacalcet and 35.7% Etelcalcetide. Those opting for a combined treatment involving both a vitamin D analog and a calcimimetic constituted 22.6% of the total (Table 1). Based on laboratory data, the mean parathyroid hormone level was 291 ± 256 pg/mL, the mean serum calcium level was 8.83 ± 0.62, and the mean serum phosphorus level was 5.44 ± 1.66. Among the included patients, 13.1% underwent parathyroidectomy. None of these data were significantly associated with fragility fractures or chronic kidney disease (CKD) data, as these values typically undergo significant changes due to continuous correction during pharmacological therapy and dialysis treatment.

Each patient suffered either a single fracture or multiple occurrences of fractures. In terms of fracture localization, 17.9% affected the upper limbs, with 6.0% in the proximal humerus, 7.1% in the humerus (other localizations), 6.0% in the distal radius, 2.4% in the radius/ulna (other localizations), and 1.2% in the hand (carpals, metacarpals, and phalanx). Lower limbs were affected in 13.1% of patients, with 2.4% affecting the proximal femur, 1.2% affecting the tibia, 3.6% being ankle fractures, and 10.7% being foot fractures. For other regions, pelvic ring fractures accounted for 2.4%, and acetabular fractures accounted for 1.2%. 

In a comparison of fracture rates among different groups of patients distinguished by the treatment they underwent, there were no significant results, except for proximal femur fractures. In this case, patients undergoing combined therapy with both a calcimimetic and a vitamin D analog seem to have a significantly lower number of proximal femur fractures (*p*-value: 0.008 *) (Table 2).

**Figure 1:**
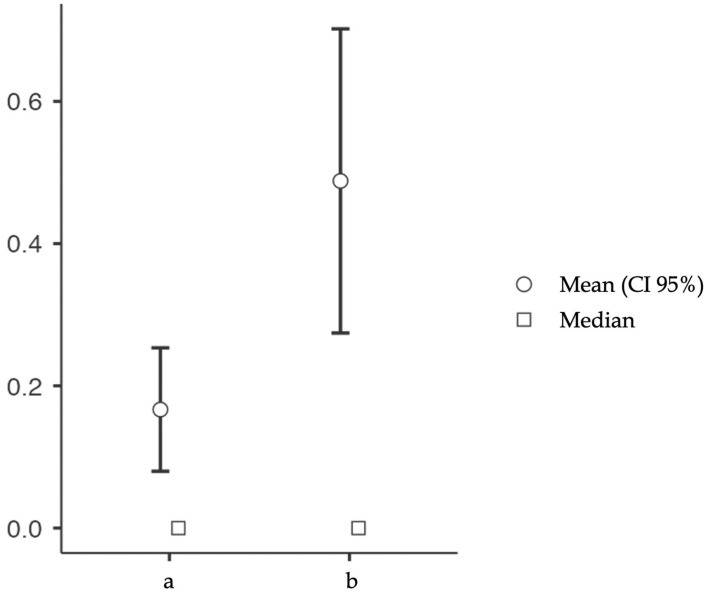
The number of fractures before (a) and after (b) CKD diagnosis is depicted in the graph. This graphic illustrates how the number of fractures increased after the diagnosis (*p*-value: 0.004).

After determining the number of fractures before (a) and after (b) CKD diagnosis for each patient, we conducted a paired t-test to examine the significance of the hypothesis b > a, indicating whether CKD significantly impacts fracture occurrence. The analysis revealed that there was a significantly higher number of fractures after CKD diagnosis (*p*-value: 0.004 *) (Figure 1). 

Logistic regression analysis, adjusted for age, sex, and CCI, revealed that smoking habits play a significant role as a risk factor for fragility fractures in the lower limbs among end-stage CKD patients (*p*-value: 0.011 *), particularly in the case of ankle fractures (*p*-value: 0.046 *). Additionally, there is a trend towards significance for foot fractures (*p*-value: 0.052) (Table 3).

**Figure 2:**
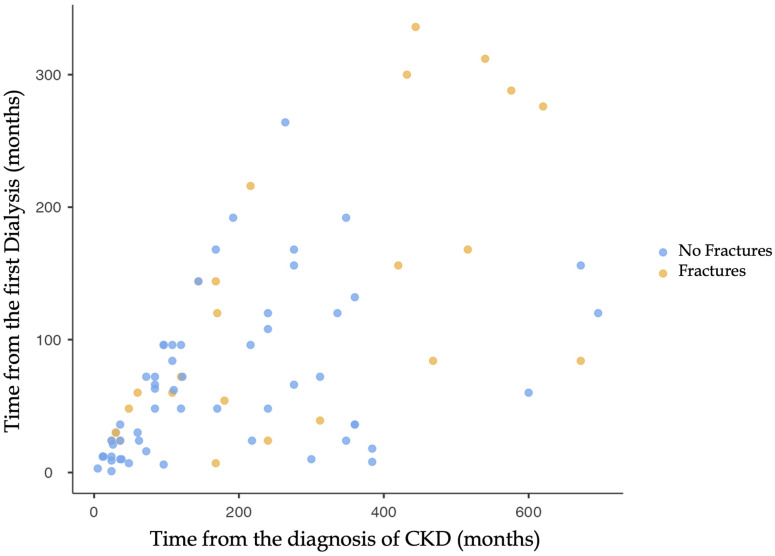
The distribution of patients according to the time since the diagnosis of CKD (*x*-axis) and the time since the first dialysis treatment (*y*-axis) is depicted in the graph. Patients with fragility fractures are highlighted with a different color from those without fractures. The graphic illustrates how the distribution of fragility fractures increases directly proportional to the time from diagnosis (*p*-value: 0.021) and the beginning of dialytic treatment (*p*-value: 0.001).

In contrast, other risk factors, such as diabetes, Charlson Comorbidity Index score, age, and sex, did not show significance in the univariate analysis (*p*-value > 0.05). However, when examining CKD-related data, the time since chronic kidney disease diagnosis, and the time since onset of dialytic treatment emerged as significant factors. Fragility fractures demonstrated a direct proportional increase concerning the time from CKD diagnosis (*p*-value: 0.021 *) to the initiation of dialytic treatment (*p*-value: 0.001 *). The latter remains significant when evaluating upper-limb (*p*-value: 0.005 *) and lower-limb (*p*-value: <0.001 *) fractures in detail (Figure 2). A logistic regression, adjusted for age and sex, was conducted to assess the impact of the time since CKD diagnosis and the time since the initiation of dialytic treatment on fragility fractures, mitigating potential biases attributed to age and sex. Notably, significant results were observed in patients with more than 72 months from the diagnosis (*p*-value: 0.041 *) and in those with more than 24 months from the commencement of dialysis (*p*-value: 0.046 *). (Table 4). 

## 4. Discussion

The presented study explores fragility fractures among patients diagnosed with end-stage chronic kidney disease (CKD), offering a perspective on the observed correlations and potential implications of several risk factors and treatments in relation to the development of fragility fractures. The intricate relationships of different factors, both patient-specific and CKD-related, were analyzed and assessed using different types of data collected retrospectively.

The demographic profile of the cohort, characterized by a mean age of 64.3 years and a predominant male proportion (58.3%), is in line with the typical age distribution seen in CKD populations [21,22]. This study also highlights the well-known extensive chronicity of CKD, with an average duration of 208 months from diagnosis, reflecting the long-term impact on skeletal health. Furthermore, the mean duration of dialytic treatment is significant regarding its average values and impact on the rate of fragility fractures. A noteworthy facet is the diverse spectrum of treatment modalities employed for mineral bone disease, generally administered through a predominant use of vitamin D analogs and calcimimetics, according to nephrological guidelines [23,24]. The observed association between combined therapy (a calcimimetic and a vitamin D analog) and a reduced number of proximal femur fractures introduces an interesting inspiration for further investigation. This is particularly significant considering the pivotal role that hip fractures play in the daily routines of trauma surgeons, especially with respect to the elderly. The associated healthcare costs and consequences for patients are considerable, contributing to remarkably high levels of morbidity and mortality. The potential synergistic effects of these treatments on bone health need, however, further exploration, with considerations made for underlying mechanisms [25,26].

The logistic regression analysis, adjusted for confounding variables such as CCI, age, and sex, identified smoking habits as a significant risk factor for fragility fractures in the lower limbs among end-stage CKD patients. This finding raises interesting questions about the underlying pathophysiological mechanisms linking smoking to skeletal fragility [27,28]. Possible explanations may involve the well-established impact of smoking on vascular health, potentially compromising bone perfusion and integrity. This discovery is considered novel as smoking habits have not been commonly included among the recognized risk factors in the existing literature [29,30]. 

This study’s emphasis on temporal factors, namely, the time since CKD diagnosis and the initiation of dialytic treatment, adds a time-related factor to fracture risk assessment. The notable associations identified, especially in the context of upper- and lower-limb fractures, indicate a deteriorating trend in bone health as CKD progresses. As depicted in Graphic 1, an extended period since the initiation of dialysis is associated with an increased risk of fracture occurrence. Notably, there is no existing literature providing data on the influence of time-related factors on the relationship between CKD and fragility fractures. This is noteworthy because the more advanced the CKD, the less effective medical therapies tend to be.

These findings underscore the need for tailored interventions addressing specific risk factors, with smoking cessation programs emerging as a potential target for fracture prevention strategies in this population. The observed variations in fracture risk based on treatment modalities highlight the complex balancing required in managing CKD-related mineral bone disorders [23]. Another option is to promote more rigorous follow-ups for patients undergoing long-term dialytic treatment. This involves assessing markers of mineral bone disease and implementing an improved treatment strategy to proactively prevent fractures.

As with any study, certain limitations need consideration, such as the retrospective nature of this study and the specific demographic characteristics of the studied cohort. The retrospective nature of this study introduces inherent limitations, such as selection bias and potential recall bias. Prospective studies would offer more solid evidence of causality and temporal relationships. The evaluation of smoking habits relies on self-reporting, potentially making this study susceptible to recall bias. Objective measures could enhance the accuracy of smoking status assessment. Additionally, many of the data trend toward significance, suggesting that these trends might potentially reach statistical significance in a larger population.

The advantages of this study are (i) the benefits of a longitudinal design, allowing for the examination of temporal relationships between CKD progression, dialysis initiation, and fragility fractures; (ii) the use of a multifactorial analysis, adjusting for age, sex, and Charlson Comorbidity Index, enriches this study’s solidity by accounting for potential confounding variables; and (iii) the fact that it contributes to the existing literature on CKD and fragility fractures, addressing a critical knowledge gap in the field and presenting findings that have not been previously observed.

## 5. Conclusions

This study enriches our understanding of fragility fractures in end-stage CKD patients, governed by a complex interplay of factors. Among patient-related factors, smoking habits seems to significantly affect lower-limb fracture rates (*p* < 0.05). Alternatively, among CKD-related factors, the time since CKD diagnosis and the time since the beginning of dialytic treatment are directly related to higher risks of fragility fractures. No relevant correlations emerged from the treatments, except for a reduction in proximal femur fracture occurrences when patients underwent a combined treatment of a calcimimetic and a vitamin D analog.

## Figures and Tables

**Table 1:** Demographic data.

**Patients (n)**	84
Male %	58.3%
Age (mean ± SD)	64.3 ± 15.2
Smoking habits (%)	19.5%
Diabetes (%)	28%
Charlson Comorbidity Index	3.06 ± 1.21
Time since CKD diagnosis (months)	208.0 ± 183.0
Time since first dialysis treatment (months)	84.5 ± 80.2
**Laboratory Data**	
PTH (pg/mL)	291 ± 256
Calcium (mg/dL)	8.83 ± 0.62
Phosphorus (mg/dL)	5.44 ± 1.66
**Treatment**	
**Vitamin D analog**	61.9%
Calcitriol	38.1%
Pericalcitol	23.8%
**Calcimimetic**	36.9%
Etelcalcetide	35.7%
Cinacalcet	1.2%
**Both treatments**	22.6%

**Table 2:** Fracture localization in different treatment groups.

Fracture Per Localization (%)	Medical Treatment for Mineral Bone Disease CKD-Related						
	Calcitriol	Paricalcitol	Vit. D Analog	Etelcalcetide	Cinacalcet	Calcimimetic	Both Treatment
**Upper limb**	8.3%	4.8%	13.1%	4.8%	0.0%	4.8%	2.4%
Proximal humerus	2.4%	2.4%	4.8%	1.2%	0.0%	1.2%	1.2%
Humerus (other)	4.8%	1.2%	6.0%	1.2%	0.0%	1.2%	1.2%
Distal radius	2.4%	1.2%	3.6%	2.4%	0.0%	2.4%	0.0%
Radius/Ulna	1.2%	1.2%	2.4%	0.0%	0.0%	0.0%	0.0%
Hand	0.0%	1.2%	1.2%	0.0%	0.0%	0.0%	0.0%
**Lower limb**	3.6%	6.0%	9.5%	6.0%	1.2%	6.0%	4.8%
Proximal femur	1.2%	1.2%	2.4%	2.4%	0.0%	2.4%	2.4% *
Femur (other)	0.0%	0.0%	0.0%	0.0%	0.0%	0.0%	0.0%
Tibia	0.0%	0.0%	0.0%	0.0%	0.0%	0.0%	0.0%
Ankle	2.4%	0.0%	2.4%	0.0%	0.0%	0.0%	0.0%
Foot	3.6%	4.8%	8.3%	3.6%	1.2%	3.6%	2.4%
**Pelvic ring**	0.0%	0.0%	0.0%	0.0%	0.0%	0.0%	0.0%
**Acetabulum**	0.0%	0.0%	0.0%	1.2%	0.0%	0.0%	0.0%

*—statistically significant (*p*-value: 0.008).

**Table 3:** Logistic regression, adjusted for age, sex, and Charlson Comorbidity Index, underscored the significant impact of smoking habits on fragility fracture rates, particularly in the lower limbs. Data are significant, especially in relation to ankle fractures, showing a trend toward significance with respect to foot fractures. Other fracture sites, both in lower and upper limbs, were not significant according to logistic regression (*p*-value > 0.05).

Variable	Stima	SE	Z	*p*
Lower-limb fractures	2.0277	0.8002	2.534	0.011 *
Ankle fractures	3.0606	1.5337	1.9956	0.046 *
Foot fractures	1.5022	0.7738	1.9414	0.052

*—statistically significant (*p*-value < 0.05).

**Table 4:** Logistic regression, adjusted for age and sex, underscored the significant impact of time since the diagnosis of CKD and, in particular, the time since the first dialysis on fragility fracture rates. Data results are significant for patients who had been diagnosed more than 72 months ago and for those whose dialytic treatment had been initiated more than 24 months prior (*p*-value < 0.05).

Variable	Stima	SE	Z	*p*
>72 months from Diagnosis	1.6650	0.8362	1.99	0.041 *
>24 months from first Dialysis	1.4183	0.6925	2.048	0.046 *

* statistically significant (*p*-value < 0.05).

## Data Availability

The datasets used and/or analyzed during the current study are available from the corresponding author on reasonable request.
